# Association genetics of chilling injury susceptibility in peach (*Prunus persica* (L.) Batsch) across multiple years

**DOI:** 10.1007/s13205-012-0109-x

**Published:** 2013-01-05

**Authors:** Arun Prabhu Dhanapal, Carlos H. Crisosto

**Affiliations:** 1 Department of Plant Sciences, University of California Davis, One Shields Ave, Davis, CA 95616 USA; 2 Present Address: Division of Plant Sciences, University of Missouri, 1-31 Agricultural Building, Columbia, MO 65211 USA

**Keywords:** Chilling injury (CI), Association analysis, Peach and SNPs

## Abstract

Peach and nectarine (*Prunus persica* L.) are highly perishable; they ripen and deteriorate quickly at ambient temperature. Storage at low temperature (0–5 °C) is a common strategy used to slow the ripening processes and to extend shelf life. However, if susceptible varieties are held too long at a low temperature, they will not ripen properly and will develop chilling injury (CI) symptoms like mealiness (M), flesh browning (FB), and flesh bleeding (FBL). Understanding the genetic control of these traits to produce CI resistant cultivars will greatly benefit producers, shippers and consumers. In this study, we evaluated a population of 51 individuals from Pop-DG across 4 years with CI traits observed in one or two time points to detect molecular marker association with selected 960 single-nucleotide polymorphisms (SNPs) from 1,536 SNPs chip. Genotypic and phenotypic data were analyzed by general linear model and mixed linear model to see comparative results from both analyses. Among 960 SNPs used, 22 SNPs were found associated with CI susceptibility traits like M, FB, and FBL. Many SNP markers were located in or close to previously reported quantitative trait loci mapped by linkage analysis.

## Introduction

Peach tree (*Prunus persica*) is a species of Prunus, a genus that also includes nectarine, plum, apricot, cherry, and almond belonging to the subfamily Prunoideae of the family Rosaceae. It is considered one of the genetically most well characterized species in the Rosaceae, and it has distinct advantages that make it suitable as a model genome species for Prunus as well as for other species in the Rosaceae (Abbott et al. 2002; Shulaev et al. 2008). Peach is a diploid with *n* = 8 and has a comparatively small genome currently estimated to be ~220–230 Mbp based upon the peach v1.0 assembly. Peach has a relatively short juvenility period of 2–3 years compared with most other fruit tree species that require 6–10 years. Peach and nectarine (*Prunus persica* L.) are highly perishable; they ripen and deteriorate quickly at ambient temperature. Storage at low temperature (0–5 °C) is a common strategy used to slow the ripening processes and to extend shelf life. Susceptibility of stone fruit to chilling injury (CI) is highly influenced by the genetic background of the cultivar (Peace et al. 2006). The physiological basis of CI symptoms has been studied in detail and well reviewed in peach (Lurie and Crisosto 2005). However, the exact mechanism by which CI affects a commodity is not fully understood. It has been shown to involve loss of membrane integrity and ion leakage from cells and changes in enzyme activity (Brummell et al. 2004a, b), but exactly why some crops are susceptible and some resistant still remains unclear.

Understanding the genetic control of these traits, to grow only cultivars free of CI susceptibility, promises to greatly benefit producers, shippers, and consumers in the peach industry. The major symptoms of CI are flesh mealiness (M), flesh browning (FB), and flesh bleeding (FBL) (Crisosto et al. 1999). M is a fruit flesh textural disorder, where affected ripe fruit have a dry, grainy feel when chewed. In simple terms, mealy fruit are dry and soft when ripe (Ju et al. 2000). FB is often seen in mealy fruit, although it can also occur in the absence of mealiness (Crisosto et al. 1999). It occurs when enzymes such as polyphenol oxidase act on phenolic substrates with which they come in contact. FBL is caused by the movement of water-soluble red pigments, probably anthocyanins, through the fruit flesh during cold storage or after subsequent ripening (Lurie and Crisosto 2005).

In the last decade, several linkage maps obtained using molecular markers have been constructed for peach (Aranzana et al. 2002; Dirlewanger et al. 2006; Etienne et al. 2002). A consensus map from an inter-specific almond × peach F2 population (‘Texas’ × ‘Earlygold’, T × E) is considered the reference map of the Prunus genus (Howad et al. 2005). However, many important agronomic characters of Prunus species have not yet been mapped, and very few of those already mapped (such as major genes for disease and pest resistances, self-incompatibility, and several fruit quality traits such as flesh color, endocarp staining, flesh adherence to stone, non-acid fruit, skin pubescence, skin color, and fruit shape) are currently being used for marker-assisted selection (MAS) (Dirlewanger et al. 2004). The application of next generation sequencing technologies and bioinformatic scripts to generate high frequency single nucleotide polymorphisms (SNPs) distributed throughout the peach genome for use in genome mapping and phenotype selection and development of high density genetic linkage maps using SNP markers were considered for two breeding populations, Pop-DF (‘Dr. Davis’ × ‘F8, 1-42’) with 117 progeny and Pop-DG (‘Dr. Davis’ × ‘Georgia Belle’) with 64 progeny (Martínez-García et al. 2012). New, integrated, and saturated SNP linkage map and high density QTL discovery in our previous study and marker-trait association across 4 years in our present one will be a valuable resource for researchers working in peach and other related species. Transcriptomic analyses of two peach cultivars namely Oded and Hermoza, which differ in their resistance to CI, were examined after 2 weeks of cold storage at 5 °C using ChillPeach cDNA microarray platform and identified 107 CGs proposed to be involved in CI (Dagar et al. 2012).

Genome-wide association analysis (GWAS) is a powerful approach to identify the causal genetic polymorphisms underlying complex traits (Riedelsheimer et al. 2012; Zhao et al. 2011). Development of genomics has provided alternative tools to improve breeding efficiency in plant breeding programs. Molecular markers linked to the causal genes and/or quantitative trait loci (QTLs) can be used for MAS (Xu and Crouch 2008). The key constraint for the successful use of association analysis in plants is the population structure and genetic relatedness, which can result in spurious marker-trait associations that may make it difficult to distinguish loci that truly affect the target traits (Chan et al. 2011; Ersoz et al. 2007; Gupta et al. 2005). Recent advances in genome sequencing and SNP genotyping have increased the applicability of association analysis for QTL mapping in crops (Morgante and Salamini 2003; Rafalski 2010).

SNPs have a low mutation rate and are evolutionarily stable from generation to generation across the genome (Batley and Edwards 2009). SNP markers have several advantages for genetic mapping over other molecular markers. SNPs have fewer detection/evaluation errors than simple sequence repeats (SSRs) (Hamblin et al. 2007) and map QTLs with greater precision than is possible with restriction fragment length polymorphisms (RFLPs) or SSRs (Yu et al. 2011). SNPs are often transferable across species within a genus (Grattapaglia et al. 2011). The development of a 6,654 peach SNP panel from which a 1,536 SNP set was selected for our previous study (Ahmad et al. 2011). Single nucleotide polymorphism (SNP) markers covering the entire genome are needed to enable molecular breeding efforts such as genome-wide association studies, fine mapping, genomic selection, and MAS in peach [*Prunus persica* (L.) Batsch] and related *Prunus* species. To date only a limited number of genetic markers, including SSRs have been available, this issue was additionally addressed by an international consortium (The International Peach SNP Consortium; IPSC) in a coordinated effort to perform genome-scale SNP discovery in peach using next generation sequencing platforms to develop and characterize a high-throughput Illumina Infinium^®^ SNP genotyping array platform (Verde et al. 2012).

In previous studies, both major and minor QTLs controlling mealiness, browning, and bleeding were localized, using phenotypic data collected for three harvest seasons. The endopolygalacturonase gene, at the *F*-*M locus*, is responsible for a major QTL controlling both mealiness and bleeding, while one of the minor QTLs for bleeding was located close to the flesh color (Ogundiwin et al. 2007). QTLs for mealiness, graininess, leatheriness, and bleeding were localized on the LG4 of V × BT population and a major QTL for mealiness was validated in V × BT population and QTLs for browning were not found on LG4 in our previous work (Cantín et al. 2010). Our previous study resulted in first genetic linkage map of CI susceptibility in peach with SSR and SNP markers (Dhanapal et al. 2012). The aim of this study was to identify SNPs association with CI susceptibility in peach across multiple years. This study will represent the most complete and comprehensive association analysis to date of CI symptoms in Pop-DG progeny populations and contributes to our present understanding of the genetic control of CI symptoms in peach. Strong marker-trait association was detected for M, FB, and FBL making breeding for resistance or low susceptibility to CI an achievable goal. New, integrated, and saturated SNP linkage map and high density QTL discovery in our previous study and marker-trait association across 4 years in our present one will permit development of new markers to aid selection of new cultivars with low susceptibility or resistance to CI. To our knowledge this is the first GWAS in peach using SNP markers for CI across 4 years with one or two time points.

## Materials and methods

### Plant materials, DNA isolation, and quantification

‘Pop-DG’, a peach intraspecific cross between ‘Dr. Davis’ (female parent) and ‘Georgia Belle’ (pollen parent), was used in this study. ‘Dr.Davis’ is a modern canning peach cultivar and ‘Georgia Belle’ is a century-old fresh market peach cultivar which contrasts for many fruit quality and other CI related traits. ‘Pop-DG’ was created and managed at Kearney Agricultural Center (Parlier, CA, USA). This orchard was established in 1998 containing 51 verified hybrids. Each progeny genotype was represented by two trees in the orchard; the leaves were collected from parents and 51 Pop-DG populations, any one of the orchard tree and frozen in liquid nitrogen and stored at −80 °C until used. Peach DNA was extracted from new leaves by the CTAB method (Doyle and Doyle 1987), modified by the addition of 1 % 2-mercaptoethanol to the isolation buffer. A preliminary quantification of DNA was determined after electrophoresis on 1 % agarose gels by comparison against a λ *Hin*dIII standard of known concentration (Peace et al. 2005) (note: the GoldenGate^®^ Genotyping assay requires using at least 15 μl of 50 ng/μl DNA per sample). The samples were sent to the DNA Technologies Core at the UC Davis Genome Center (http://dnatech.genomecenter.ucdavis.edu/dna_quant.html), and a second DNA quantification was done on a Molecular Devices Analyst plate reader with Pico Green (Invitrogen Molecular Probes). Phenotypic evaluation was carried out similar to our previous work (Ogundiwin et al. 2007). For genotyping of Pop-DG, five separate DNA samples of “Dr. Davis” and two “Georgia Belle” samples were used.

### Discovery and selection of SNPs

The advantage of association mapping is that it can map quantitative traits with high resolution in a way that is statistically very powerful. Association mapping, however, also requires extensive knowledge of SNPs within the genome of the organism of interest, and is therefore difficult to perform in species that have not been well studied or do not have well-annotated genomes. In our present study, a set of 6,654 high-quality SNPs was developed under the framework of the “Integration of genomic tools for next generation peach and almond cultivar development” USDA-NRI project at UC Davis. SNPs were obtained by generation of whole genome sequence for “Dr. Davis”, “F8, 1-42” and “Georgia Belle” using both Roche 454 and Ilumina-Solexa technologies (Ahmad et al. 2011). Assembly and alignment were done according to our previous published work (Ahmad et al. 2011). The SNPs were selected to be evenly distributed across the genome from a larger SNP set of 6,654 peach SNPs (approximately 1 SNP/40,000 nucleotides). SNPs for Pop-DG, a large number of these SNPs were found to be heterozygous for both parents, providing 1:2:1 segregation ratios. SNPs were also evaluated with a “design” score generated by Illumina, which attempts to predict the probability of success when used in a GoldenGate^®^ assay. SNPs with scores = 1.1 were used for mapping. SNPs were named starting with “UCD” for the University of California, Davis, followed by “SNP” for the marker type and the number of their order in the OPA (UCD_SNP_XX). Once the final SNPs were selected, their reproducibilities and heritabilities were examined for Pop-DG population. Linkage group (LG) was constructed from final set of most informative markers and individuals from Pop-DF and Pop-DG population (Martínez-García et al. 2012). SNP descriptions are provided in supplemental files 1 and 2 (Martínez-García et al. 2012), referenced to the SNP entries in the NCBI SNP database in the range NCBIdbSNP:275372743 to NCBI-dbSNP:275395485.

### Oligonucleotide pool (OPA) development

Oligonucleotides were designed, synthesized, and assembled into this OPA by Illumina Inc. The peach “GS0012410-OPA.opa” consisted of 1,536 SNPs and was used to genotype the Pop-DF and Pop-DG populations (Martínez-García et al. 2012). This is a unique set of SNP oligonucleotides developed for our previous project, but could be applied to any number of peach mapping/evaluation projects (Ahmad et al. 2011). For our present study, 1,536 SNPs genotyped from “Dr. Davis” and “Georgia Belle” were used. 960 SNPs with Minor Allele Frequency (MAF ≥10) were used for further analysis.

### SNP genotyping

**Fig. 1:**
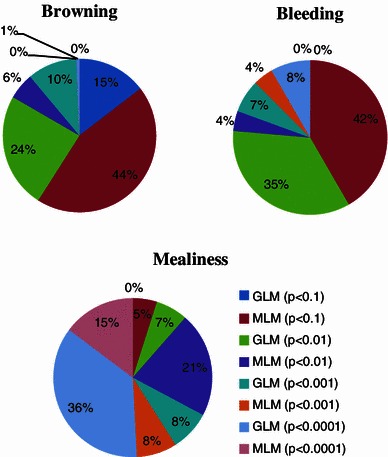
*Pie charts* depicting SNP-trait association for browning, bleeding, and mealiness in two different analyses namely GLM and MLM based on various levels of significance

The SNPs were evaluated in an Illumina GoldenGate^®^ Genotyping assay (Fan et al. 2003) with the iScan readout at the UC Davis Genome Center. The results were analyzed with Genome Studio™ Genotyping Module v1.0 from Illumina (Fig. 1). The SNPs were manually edited and removed from the analysis if clustering (segregation) errors in the parents/progeny distributions were observed, as described in the Infinium^®^ Genotyping Data Analysis from Illumina^®^Technical Note (http://www.illumina.com), except that the Gen-Call score threshold level for inclusion of data into clusters was changed from 0.15 to 0.25 (Higher Gen Call scores indicate that the data points are more reliable and can be included in a specific cluster with more confidence). The deviations from expected allelic Mendelian inheritance ratios at all loci for each progeny were measured according to our previous paper (Martínez-García et al. 2012).

### Association analysis

The population structure was inferred using the software program STRUCTURE 2.2 (Pritchard et al. 2000) on 960 SNPs (MAF ≥10 %) as described (Hao et al. 2012). In brief, five independent iterations of running were performed with hypothetic number of subpopulation (*k*) ranging from 1 to 10, based on burn-in period and the number of Markov chain Monte Carlo (MCMC) replications after burn-in, which were all assigned at 100,000. The correct estimation of *k* was provided by joining the log probability of data [Ln *P*(*D*)] from the STRUCTURE output and an ad hoc statistic Δ*k* (Evanno et al. 2005), which was based on the rate of change in the log probability of data between successive *k* values. Based on *k* = 2 all 51 progenies were assigned subpopulation. The population structure matrix (*Q*) was generated for further analysis. To account for the population structure, four statistical models were evaluated: the general linear model (GLM) model without considering *Q* and *K*, the Q model with considering *Q* (not shown), the K model with considering *K* (not shown) and mixed linear model (MLM) model considering *Q* and *K* (Yu and Buckler 2006). Results of only two models, GLM model without considering *Q* and *K* and MLM model considering *Q* and *K*, were shown. Genome-wide association analyses based on these models were conducted with the software TASSEL 2.1. (Buckler et al. 2009) Markers were defined as being significantly associated with traits on the basis of their significant association threshold (−Log *P* ≥ 2.00, *P* < 0.0001, *P* < 0.001, *P* < 0.01 and *P* < 0.1). (Buckler et al. 2009; Ge et al. 2003).

## Results and discussion

### Genome-wide association analysis (GWAS)

GWAS have been more successful in crop plants than in humans (Brachi et al. 2011). GWAS overcomes several limitations of traditional gene mapping by (1) providing higher resolution, often to the gene level, and (2) using samples from previously well-studied populations in which commonly occurring genetic variations can be associated with phenotypic variation (Brachi et al. 2011). Using two models namely GLM and MLM, associations between CI traits and 960 SNPs genotypes were evaluated in our studied Pop-DG population. 22 SNPs were identified to have significant marker-trait associations at different threshold levels (−Log *P* ≥ 2.00, *P* < 0.0001, *P* < 0.001, *P* < 0.01 and *P* < 0.1) for CI traits. The two models were compared based on different levels of significance across years at one or two time points. GLM analysis uses a Σ-restricted model that is a good test of marker effects, even when the data are unbalanced (Bradbury et al. 2007; Buckler et al. 2009). Similar to GLM, MLM performs an association test for each combination of traits and markers. 960 SNPs (MAF ≥10 %) were tested for association with CI traits like M, FB, and FBL. Results were observed and reported at four different significance levels with both models. Only SNPs with minimum significance level (*P* < 0.1) at least 3 years were considered without considering the time points.

### Mealiness (M)

Among 22 SNPs that showed association for different CI traits across 4 years, only two SNPs were synchronously associated with M and FBL at one or both time points in all 4 years. Both SNPs (UCD_SNP_1084 and UCD_SNP_1441) were on chromosome 4 (Table 4). For M, UCD_SNP_1084, UCD_SNP_1441, and UCD_SNP_1172 were identified in 3 years at both time points with following significance level (GLM, *P* < 0.0001; MLM, *P* < 0.01 or *P* < 0.001). Significance levels observed in both time points in year 2007 were less when compared with other 3 years. All four SNPs that showed association for M were on chromosome 4 (Table 1). For M, proportion of higher level of significance (36 %) was found in GLM model (*P* < 0.0001) followed by MLM model (*P* < 0.01). Three SNPs namely UCD_SNP_1084, UCD_SNP_1441 and UCD_SNP_1172 should have contributed for higher significance levels in both models (Fig. 1). QTLs identified for mealiness (qML1; LOD score 4.18) at 51.06 cM in LG1 was close to previously mapped CG19 (candidate gene) at 52.0 cM with flanking marker UCD_SNP_297. Another QTL for mealiness (qML4.1; LOD score 8.74) at 35.89 cM in LG4 was 6.6 cM away from CG36 (Dhanapal et al. 2012; Martínez-García et al. 2012). Three SNPs (UCD_SNP_1084, UCD_SNP_1441, and UCD_SNP_1172) were exactly on same position on LG4, where qML4.1 was found.

**Table 1:** GLM and MLM analysis across 4 years showing significant SNP marker associated for mealiness in one or two different time points

SNP name	Mealiness 2002		Mealiness 2003				Mealiness 2004		Mealiness 2007			
	3 weeks		2 weeks		3 weeks		3 weeks		2 weeks		3 weeks	
	GLM	MLM	GLM	MLM	GLM	MLM	GLM	MLM	GLM	MLM	GLM	MLM
UCD_SNP_56	3.85E−05***	2.25E−03*	7.27E−08***	2.32E−04*	1.50E−10***	4.02E−06***	5.34E−10***	4.79E−06***	3.39E−05***	1.21E−03*	2.86E−09***	8.93E−04**
UCD_SNP_1084	1.60E−06***	8.05E−04**	1.38E−07***	3.20E−05***	3.78E−10***	5.10E−06***	2.30E−09***	7.27E−06***	7.01E−06***	1.82E−03*	1.28E−09***	1.79E−03*
UCD_SNP_1441	1.60E−06***	8.05E−04**	1.38E−07***	3.20E−05***	3.78E−10***	5.10E−06***	2.30E−09***	7.27E−06***	7.01E−06***	1.82E−03*	1.28E−09***	1.79E−03*
UCD_SNP_1172	2.56E−05***	2.25E−03*	2.52E−06***	1.66E−04**	1.86E−08***	4.47E−05***	1.40E−06***	1.85E−04**	1.20E−04**	3.70E−03*	2.32E−04**	4.45E−03*

2 weeks, measurement taken at room temperature after 2 weeks of cold storage at 5 °C; 3 weeks, measurement taken at room temperature after 3 weeks of cold storage at 5 °C

* Significance at *P* < 0.01; ** significance at *P* < 0.001; *** Significance at *P* < 0.0001

**Table 2:** GLM and MLM analysis across 4 years showing significant SNP marker associated for browning in one or two different time points

SNP Name	Browning 2002		Browning 2003				Browning 2004		Browning 2007			
	3 weeks		2 weeks		3 weeks		3 weeks		2 weeks		3 weeks	
	GLM	MLM	GLM	MLM	GLM	MLM	GLM	MLM	GLM	MLM	GLM	MLM
UCD_SNP_1422	1.79E−05***	4.12E−03*	2.90E−03*	3.28E−02^‡^	2.30E−04**	1.34E−02^‡^	3.38E−03*	4.72E−02^‡^	1.06E−04**	2.37E−02^‡^	6.64E−03*	2.30E−02^‡^
UCD_SNP_211	4.82E−04**	5.92E−03*	4.00E−03*	4.62E−02^‡^	8.30E−03*	3.18E−03*	2.53E−02^‡^	4.29E−02^‡^	1.00E−02^‡^	4.08E−02^‡^	4.89E−02^‡^	9.44E−02^‡^
UCD_SNP_234	4.82E−04**	5.92E−03*	4.00E−03*	4.62E−02^‡^	8.30E−03*	3.18E−03*	2.53E−02^‡^	4.29E−02^‡^	1.00E−02^‡^	4.08E−02^‡^	4.89E−02^‡^	9.44E−02^‡^
UCD_SNP_284	9.12E−04**	1.43E−02^‡^	7.83E−03*	1.22E−02^‡^	6.35E−03*	4.01E−02^‡^	1.55E−02^‡^	4.96E−02^‡^	3.67E−02^‡^	4.43E−02^‡^	2.02E−02^‡^	7.02E−02^‡^
UCD_SNP_751	4.82E−04**	5.92E−03*	4.00E−03*	6.62E−03*	1.64E−03*	3.18E−02^‡^	2.53E−02^‡^	4.29E−03*	1.00E−02^‡^	4.08E−02^‡^	4.89E−02^‡^	9.44E−02^‡^
UCD_SNP_931	9.12E−04**	1.43E−02^‡^	7.83E−03*	1.22E−02^‡^	6.35E−03*	4.01E−02^‡^	1.55E−02^‡^	4.96E−02^‡^	3.67E−02^‡^	4.43E−02^‡^	2.02E−02^‡^	7.02E−02^‡^
UCD_SNP_1219	9.19E−04**	3.43E−02^‡^	4.89E−03*	2.92E−02^‡^	7.01E−04**	1.13E−02^‡^	1.49E−03*	1.35E−02^‡^	2.61E−03*	4.18E−02^‡^	3.06E−03*	5.01E−02^‡^
UCD_SNP_1457	9.20E−04**	3.41E−02^‡^	4.70E−03*	2.84E−02^‡^	6.35E−04**	1.08E−02^‡^	1.50E−03*	1.45E−02^‡^	2.61E−03*	4.17E−02^‡^	7.43E−03*	1.09E−02^‡^
UCD_SNP_1460	5.97E−04**	2.03E−02^‡^	1.17E−03*	1.17E−02^‡^	1.15E−03*	2.99E−02^‡^	1.04E−02^‡^	2.09E−02^‡^	4.53E−02^‡^	4.49E−02^‡^	3.26E−02^‡^	5.33E−02^‡^
UCD_SNP_3	4.74E−04**	4.27E−02^‡^	1.96E−03*	2.29E−02^‡^	7.00E−03*	1.92E−02^‡^	5.23E−03*	1.79E−02^‡^	4.00E−02^‡^	3.33E−02^‡^	4.80E−03*	1.27E−02^‡^
UCD_SNP_108	4.74E−04**	4.27E−02^‡^	1.96E−03*	2.29E−02^‡^	7.00E−03*	1.92E−02^‡^	5.23E−03*	1.79E−02^‡^	4.00E−02^‡^	3.33E−02^‡^	4.80E−03*	1.27E−02^‡^
UCD_SNP_455	4.74E−04**	4.27E−02^‡^	1.96E−03*	2.29E−02^‡^	7.00E−03*	1.92E−02^‡^	5.23E−03*	1.79E−02^‡^	4.00E−02^‡^	3.33E−02^‡^	4.80E−03*	1.27E−02^‡^

2 weeks, measurement taken at room temperature after 2 weeks of cold storage at 5 °C; 3 weeks, measurement taken at room temperature after 3 weeks of cold storage at 5 °C

^‡^Significance at *P* < 0.1; * significance at *P* < 0.01; ** significance at *P* < 0.001; *** significance at *P* < 0.0001

**Table 3:** GLM and MLM analysis across 4 years showing significant SNP marker associated for bleeding in one or two different time points

SNP name	Bleeding 2002		Bleeding 2003				Bleeding 2004		Bleeding 2007			
	3 weeks		2 weeks		3 weeks		3 weeks		2 weeks		3 weeks	
	GLM	MLM	GLM	MLM	GLM	MLM	GLM	MLM	GLM	MLM	GLM	MLM
UCD_SNP_56	2.63E−03*	5.44E−03*	1.90E−03*	9.31E−04**	9.68E−04**	1.03E−02^‡^	8.94E−05***	1.56E−02^‡^	3.93E−03*	3.92E−02^‡^	5.00E−03*	4.70E−02^‡^
UCD_SNP_821	4.73E−04**	2.16E−02^‡^	9.05E−03*	1.71E−02^‡^	6.73E−03*	4.07E−02^‡^	8.57E−04**	4.30E−02^‡^	2.93E−03*	2.97E−02^‡^	1.00E−03*	4.18E−02^‡^
UCD_SNP_1084	1.43E−03*	3.26E−03*	7.99E−05***	5.56E−04**	7.21E−03*	4.88E−02^‡^	2.54E−05***	1.04E−02^‡^	2.78E−03*	2.80E−02^‡^	3.58E−03*	4.72E−02^‡^
UCD_SNP_1441	1.43E−03*	3.26E−03*	7.99E−05***	5.56E−04**	7.21E−03*	3.88E−02^‡^	2.54E−05***	1.04E−02^‡^	2.78E−03*	2.80E−02^‡^	3.58E−03*	4.72E−02^‡^
UCD_SNP_1507	4.73E−04**	2.16E−02^‡^	9.05E−03*	1.71E−02^‡^	6.73E−03*	4.07E−02^‡^	8.57E−04**	4.30E−02^‡^	2.93E−03*	2.97E−02^‡^	1.00E−03*	4.18E−02^‡^
UCD_SNP_1416	3.96E−03*	4.19E−02^‡^	3.21E−03*	6.24E−02^‡^	4.03E−03*	4.71E−02^‡^	3.54E−05***	1.15E−02^‡^	2.97E−03*	2.98E−02^‡^	9.86E−03*	3.54E−02^‡^

2 weeks, measurement taken at room temperature after 2 weeks of cold storage at 5 °C; 3 weeks, measurement taken at room temperature after 3 weeks of cold storage at 5 °C

^‡^Significance at *P* < 0.1; * significance at *P* < 0.01; ** significance at *P* < 0.001; *** significance at *P* < 0.0001

### Browning (FB)

Among 960 SNPs used, 12 SNPs showed association for FB. Seven SNPs were on chromosome 5 and two SNPs were on chromosome 2 (Table 4). All SNPs showed higher level of significance in 2002 for GLM analysis (*P* < 0.001). Even though large number of markers showed association for FB, level of significance was less when compared with M for all SNPs showing association. For trait FB significance levels observed in both time points in year 2007 was less when compared with other 3 years (Table 2). Higher proportion of significance (44 %) was found in MLM model (*P* < 0.1) followed by GLM model (*P* < 0.01). Trait FB did not show higher significance levels (*P* < 0.0001) of association for all 12 SNPs (Fig. 1). More than one significant SNPs associated with QTL was identified for flesh browning in LG5 (qBrL5) in previous study (Martínez-García et al. 2012). CG1 was at same position of significant marker identified (UCD_SNP_1422) for flesh browning and CG38 was 4.5 cM away from qBrL5 (Dhanapal et al. 2012). In our present association analysis one SNP, UCD_SNP_1422, was found along with 11 others SNPs that showed association for browning.

**Table 4:** SNP marker for different CI traits with their position in Pop-DG linkage map and in scaffolds of peach genome

Name of trait	Name of SNPs	Position in Pop-DG linkage map (cM)	Position in scaffolds of peach genome (Mb)
Mealiness	UCD_SNP_56	37.7	SNP_at_scaffold_4:27407656
	UCD_SNP_1084	35.9	SNP_at_scaffold_4:27275348
	UCD_SNP_1441	35.9	SNP_at_scaffold_4:27336281
	UCD_SNP_1172	39.4	SNP_at_scaffold_ 4:27680840
Browning	UCD_SNP_1422	29.6	SNP_at_scaffold_5: 9870651
	UCD_SNP_211	3.5	SNP_at_scaffold_2: 4024474
	UCD_SNP_234	3.5	SNP_at_scaffold_2: 3973961
	UCD_SNP_284	3.5	SNP_at_scaffold_2: 3627982
	UCD_SNP_751	3.5	SNP_at_scaffold_2: 3811227
	UCD_SNP_931	3.5	SNP_at_scaffold_2: 4273409
	UCD_SNP_1219	34.4	SNP_at_scaffold_5: 11252108
	UCD_SNP_1457	33.6	SNP_at_scaffold_5: 11475626
	UCD_SNP_1460	25.7	SNP_at_scaffold_5: 9357411
	UCD_SNP_3	10.8	SNP_at_scaffold_5: 4219129
	UCD_SNP_108	11	SNP_at_scaffold_5:4289138
	UCD_SNP_455	10.6	SNP_at_scaffold_5:4276700
Bleeding	UCD_SNP_56	37.7	SNP_at_scaffold_4:27407656
	UCD_SNP_821	31.1	SNP_at_scaffold_1:25513229
	UCD_SNP_1084	35.9	SNP_at_scaffold_4:27275348
	UCD_SNP_1441	35.9	SNP_at_scaffold_4:27336281
	UCD_SNP_1507	63.9	SNP_at_scaffold_1:43389449
	UCD_SNP_1416	24.3	SNP_at_scaffold_4:17093999

### Bleeding (FBL)

Analyzing data using both models, we were able to identify only six SNPs for FBL. All six were on chromosome 1 and 4 only (Table 4). Two SNPs (UCD_SNP_1084, UCD_SNP_1441) that showed association for M also showed association for trait FBL. These two SNPs were co-associated for M and FBL. Trait M showed higher level (*P* < 0.0001) of association using both models compared with FBL (GLM, *P* < 0.01; MLM, *P* < 0.01 or *P* < 0.1) (Table 3). Similar to FB, trait FBL also showed same higher proportion of significance (42 %) found in MLM model (*P* < 0.1) followed by GLM model (*P* < 0.01). Even though same SNPs (UCD_SNP_1084, UCD_SNP_1441) co-associated for trait M, level of significance observed was not similar (Fig. 1). Four QTLs identified for flesh bleeding in which three in LG1 [qBLa (17.28 cM); LOD score 3.86, qBLb (18.80 cM); LOD score 3.19, and qBLc (23.96 cM); LOD score 3.22] were close to CG14 (18.9 cM), CG5 (19.5 cM), CG16 (19.5 cM), and CG30 (19.5 cM). One QTL identified in LG4 (qBL4; LOD score 4.45) at 35.89 cM was 6.5 cM away from CG36 (Dhanapal et al. 2012; Martínez-García et al. 2012). The co associated SNPs in our present study were exactly on same position in LG4 where qBL4 was found.

### Marker-trait association for CI

Even though we used two different models in our present study, results from GLM model without considering *Q* and *K* and MLM model considering *Q* and *K* analysis did not show major difference in *P* values for all the traits under study. There was no additional markers pop out in either analysis when compared with one another but *P* values obtained from MLM analysis were lower than GLM (Tables 1, 2, 3). Two other models of GLM namely Q model with considering *Q*, the K model with considering *K* (results not shown) also resulted with same number of markers for association of various traits under study. Studies on soybean using various statistical models evaluated: the GLM model without considering *Q* and *K*; the Q model with considering *Q*; the K model with considering *K*; the MLM model with considering *Q* and *K*. MLM model performed a little better than the K model. So, the GWAS for soybean yield and yield components with the MLM model (*Q* + *K*) was used to correct for population structure and genetic relatedness, using 1,142 SNPs and 209 haplotypes (Hao et al. 2012).

In plants, QTLs were originally mapped in biparental crosses, but they were restricted in allelic diversity and in having limited genomic resolution. The advent of high-density SNP typing allowed whole-genome scans to identify often small haplotype blocks that are significantly correlated with quantitative trait variation. These approaches on plant studies have been successful in identifying loci that explain large portions of phenotypic variation, which is now available for most of the fruit tree species. The genetic control of CI related traits in peach has been studied and it has been demonstrated that mealiness, browning, and bleeding are probably controlled by major genes (Ogundiwin et al. 2007; Peace et al. 2006). Moreover, one major QTL has been detected for each of these symptoms of CI in LG4 and LG5, using a linkage map constructed from two segregating populations Pop-DG (‘Dr. Davis’ × ‘Georgia Belle’) and Pop-G (‘Georgia Belle’ selfed) (Ogundiwin et al. 2007; Peace et al. 2006).

In our previous study a major QTL for mealiness and bleeding was found at the F-M locus at the bottom end of LG4. Other minor QTLs for mealiness were also found on LG4 and LG6. Besides, an Expressed Sequence Tags (ESTs) database has been developed specifically to study CI (Ogundiwin et al. 2008). Microarray analysis involving these ESTs has identified several cold-regulated peach genes some of which have been mapped close to CI QTLs on Pop-DG (Ogundiwin et al. 2009). A gene encoding a cell wall modifying enzyme, endopolygalacturonase (endoPG) co-localized with the major QTL affecting mealiness (Callahan et al. 2004; Peace et al. 2005). Another gene in the anthocyanin biosynthesis pathway, leucoanthocyanidin dioxygenase (PpLDOX), mapped to the same genomic region where the major QTL controlling browning was identified (Ogundiwin et al. 2009). Even though several previous studies were on CI traits using molecular markers like SSRs, Restriction fragment length polymorphism (RFLP), and Single sequence conformational polymorphism (SSCP), this is the first study using SNP-based markers for CI traits.

### Genetic-based association studies in Prunus

Association mapping (AM) is one of the effective approach for connecting phenotype and genotype in plants. It enhances previous QTL information for MAS in rice (Agrama et al. 2007), wheat (Tommasini et al. 2007), and maize (Yu and Buckler 2006). Development of genetic markers is crucial for association studies. A whole genome or selected segments of a genome of crop, plants, and trees are sequenced to identify differences across the genome. Subsequently, identified polymorphisms are genotyped across a larger and more diverse yet unrelated population. Prunus fruit development, growth, ripening, and senescence all include major biochemical and sensory changes in texture, color, and flavor. The genetic dissection of these complex processes has important applications in crop improvement (Cao et al. 2012). Fan et al. (2010) constructed a linkage map on peach F2 population of 378 genotypes developed from two genotypes with contrasting chilling requirements for QTL mapping. The study detected five QTLs of chilling requirement. Among these QTLs, qCR1a and qCR7 showed very prominent effects and were declared to be the major QTLs. The two QTLs not only facilitate marker-assisted breeding for low chill cultivars, but also paved the way for future fine mapping and map-based cloning of genes controlling the chilling requirement. Another most highly significant association found was CPPCT005 on chromosome 4, which explained 25.1 % of the phenotypic variation (Fan et al. 2010).

Another study on apricot (*Prunus armeniaca*) for chilling injury requirement (CR) based on a two-way pseudo test cross mapping strategy, in which two high-density apricot maps were constructed using a total of 43 SSRs and 994 amplified fragment length polymorphism (AFLP) markers that span an average of 502.6 cM with an average marker interval of 0.81 cM. Twelve putative CR QTLs were detected using composite interval mapping, a simultaneous multiple regression fit and an additive by-additive epistatic interaction model without dominance. An average of 62.3 ± 6.3 % of the total phenotypic variance was explained. QTLs corresponding to map positions of differentially expressed transcripts and candidate genes controlling a majority of the QTLs were shown to be stable between both *Prunus* species, as well as similar trends in their QTL effects, with the allele for increasing the trait value mostly originating from the high chill parents. The comparative QTL mapping strategy presented reveals the transferability of genetic information between two *Prunus* species, the characterization of stable QTLs, the utility of the maps to consolidate each other and to further validate previously identified CR QTL as a major controlling factor driving floral bud break.

Identifying the genetic variants that underlie complex traits was very essential to plant genetics. Two main approaches are available for mapping the relevant genes and identifying the variants associated with this complex trait: linkage mapping in families and population-based genetic association studies (Agrama et al. 2007). In theory, genetic association mapping is more powerful than linkage studies in identifying variants with weak effects that may contribute risks to common complex traits (Risch and Merikangas 1996). These trait-associated SNPs from our study were compared with other reported SNPs and SSR markers from QTL mapping analyses of our previous study on selected populations. Among associations, some were in regions where QTLs associated with the given trait had previously been identified. However, some associations were not consistent with the results of other published linkage maps because of different genetic materials and different marker systems used (Cao et al. 2012).

## Conclusion

In our present study, association genetics was performed for CI susceptibility traits using GWAS based on GLM model without considering *Q* and *K* and MLM model considering *Q* and *K* analysis. A total of 22 SNPs marker-trait associations were identified across 4 year in one or two time points by analyzing both models. However, further studies are necessary to confirm the association results in specific biparental crossing populations and consequently avoid identifying spurious associations. All three SNP markers namely UCD_SNP_1084, UCD_SNP_1441 and UCD_SNP_1172 will be investigated based on their position in genome, and it would be interesting to see if these SNPs are located in regulatory region or coding region in some candidate gene of interest. The presence of considerable levels of synteny among many rosaceous species suggests that even for those plant species with fewer genomic resources, candidate-gene association mapping coupled with QTL mapping studies and comparative mapping would be feasible and highly valuable. Knowledge acquired in one species can then be extended to others. Establishing a model species and refining its genome sequence and identifying regions associated with important traits could even be used for other related species of Rosaceae by conducting comparative genomics. Our future studies include localization of genes based on SNP marker-trait association for CI traits.

## References

[CR1] Abbott AG, Lecouls AC, Wang Y, Georgi L, Scorza R, Reighard G (2002). Peach: the model genome for Rosaceae genomics. Acta Hortic.

[CR2] Agrama HA, Eizenga GC, Yan W (2007). Association mapping of yield and its components in rice cultivars. Mol Breed.

[CR3] Ahmad R, Parfitt D, Fass J, Ogundiwin E, Dhingra A, Gradziel T, Lin D, Joshi N, Martínez-García PJ, Crisosto CH (2011). Whole genome sequencing of peach (Prunus persica L.) for SNP identification and selection. BMC Genomics.

[CR4] Aranzana MJ, García-Mas J, Carbó J, Arús P (2002). Development and variability analysis of microsatellite markers in peach. Plant Breed.

[CR5] Batley J, Edwards D (2009). Mining for SNPs and SSRs using SNPServer, dbSNP and SSR taxonomy tree. Methods Mol Biol.

[CR6] Brachi B, Morris GP, Borevitz JO (2011). Genome-wide association studies in plants: the missing heritability is in the field. Genome Biol.

[CR7] Bradbury PJ, Zhang Z, Kroon DE, Casstevens TM, Ramdoss Y, Buckler ES (2007). TASSEL: software for association mapping of complex traits in diverse samples. Bioinformatics.

[CR8] Brummell DA, Dal Cin V, Crisosto CH, Labavitch JM (2004). Cell wall metabolism during maturation, ripening and senescence of peach fruit. J Exp Bot.

[CR9] Brummell DA, Dal Cin V, Lurie S, Crisosto CH, Labavitch JM (2004). Cell wall metabolism during the development of chilling injury in cold-stored peach fruit: association of mealiness with arrested disassembly of cell wall pectins. J Exp Bot.

[CR10] Buckler E, Casstevens T, Bradbury P, Zhang Z (2009). Analysis by association, evolution and linkage (TASSEL) version 2.1.

[CR11] Callahan AM, Scorza R, Bassett C, Nickerson M, Abeles FB (2004). Deletions in an endopolygalacturonase gene cluster correlate with non-melting flesh texture in peach. Funct Plant Biol.

[CR12] Cantín CM, Crisosto CH, Ogundiwin EA, Gradziel T, Torrents J, Moreno MA, Gogorcena Y (2010). Chilling injury susceptibility in an intra-specific peach [*Prunus persica* (L.) Batsch] progeny. Postharvest Biol Technol.

[CR13] Cao K, Wang L, Zhu G, Fang W, Chen C, Luo J (2012). Genetic diversity, linkage disequilibrium, and association mapping analyses of peach (*Prunus persica*) landraces in China. Tree Genet Genom.

[CR14] Chan EKF, Rowe HC, Corwin JA, Joseph B, Kliebenstein DJ (2011). Combining genome-wide association mapping and transcriptional networks to identify novel genes controlling glucosinolates in *Arabidopsis thaliana*. PLoS Biol.

[CR15] Crisosto CH, Mitchell GF, Zhiguo J (1999). Susceptibility to chilling injury of peach, nectarine, and plum cultivars grown in California. Hortic Sci.

[CR16] Dagar A, Puig CP, Ibanez CM, Ziliotto F, Bonghi C, Crisosto CH, Friedman H, Lurie S, Granell A (2012). Comparative transcript profiling of a peach and its nectarine mutant at harvest reveals differences in gene expression related to storability. Tree Genet Genomes.

[CR17] Dhanapal AP, Martinez-Garcia PJ, Gradziel T, Crisosto CH (2012). First genetic linkage map of chilling injury susceptibility in peach (*Prunus persica* (L.) Batsch) fruit with SSR and SNP markers. J Plant Sci Mol Breed.

[CR18] Dirlewanger E, Graziano E, Joobeur T, Garriga-Caldere F, Cosson P, Howad W, Arús P (2004). Comparative mapping and marker-assisted selection in Rosaceae fruit crops. Proc Natl Acad Sci USA.

[CR19] Dirlewanger E, Cosson P, Boudehri K, Renaud C, Capdeville G, Tauzin Y, Laigret F, Moing A (2006). Development of a second-generation genetic linkage map for peach [*Prunus persica* (L.) Batsch] and characterization of morphological traits affecting flower and fruit. Tree Genet Genomes.

[CR20] Doyle JJ, Doyle JL (1987). A rapid DNA isolation procedure for small quantities of fresh tissue. Phytochem Bull.

[CR21] Ersoz ES, Yu J, Buckler ES, Varshney R, Tuberosa R (2007). Applications of linkage disequilibrium and association mapping in crop plants. Genomics-assisted crop improvement.

[CR22] Etienne C, Rothan C, Moing A, Plomion C, Bodenes C, Svanella-Dumas L, Cosson P, Pronier V, Monet R, Dirlewanger E (2002). Candidate genes and QTLs for sugar and organic acid content in peach [*Prunus persica* (L.) Batsch]. Theor Appl Genet.

[CR23] Evanno G, Regnaut S, Goudet J (2005). Detecting the number of clusters of individuals using the software STRUCTURE: a simulation study. Mol Ecol.

[CR24] Fan JB, Oliphant A, Shen R, Kermani BG, Garcia F, Gunderson KL, Hansen M, Steemers F, Butler SL, Deloukas P, Galver L, Hunt S, McBride C, Bibikova M, Rubano T, Chen J, Wickham E, Doucet D, Chang W, Campbell D, Zhang B, Kruglyak S, Bentley D, Haas J, Rigault P, Zhou L, Stuelpnagel J, Chee MS (2003). Highly parallel SNP genotyping. Cold Spring Harb Sym.

[CR25] Fan SH, Bielenberg DG, Zhebentyayeva TN, Reighard GL, Okie WR, Holland D, Abbott AG (2010). Mapping quantitative trait loci associated with chilling requirement, heat requirement and bloom date in peach (*Prunus persica*). New Phytol.

[CR26] Ge Y, Dudoit S, Speed T (2003). Resampling-based multiple testing for microarray data analysis. TEST.

[CR27] Grattapaglia D, Silva OB, Kirst M, de Lima BM, Faria DA, Pappas GJ (2011). High-throughput SNP genotyping in the highly heterozygous genome of Eucalyptus: assay success, polymorphism and transferability across species. BMC Plant Biol.

[CR28] Gupta P, Rustgi S, Kulwal P (2005). Linkage disequilibrium and association studies in higher plants: present status and future prospects. Plant Mol Biol.

[CR29] Hamblin MT, Warburton ML, Buckler ES (2007). Empirical comparison of Simple Sequence Repeats and single nucleotide polymorphisms in assessment of maize diversity and relatedness. PLoS ONE.

[CR30] Hao D, Cheng H, Yin Z, Cui S, Zhang D, Wang H, Yu D (2012). Identification of single nucleotide polymorphisms and haplotypes associated with yield and yield components in soybean (*Glycine max*) landraces across multiple environments. Theor Appl Genet.

[CR31] Howad W, Yamamoto T, Dirlewanger E, Testolin R, Cosson P, Cipriani G, Monforte AJ, Georgi L, Abbott AG, Arús P (2005). Mapping with a few plants: using selective mapping for microsatellite saturation of the Prunus reference map. Genetics.

[CR32] Ju ZG, Duan JS, Ju ZQ (2000). Leatheriness and mealiness of peaches in relation to fruit maturity and storage temperature. J Hortic Sci Biotechnol.

[CR33] Lurie S, Crisosto C (2005). Chilling injury in peach and nectarine. Postharvest Biol Technol.

[CR34] Martínez-García PJ, Parfitt DE, Ogundiwin EA, Fass J, Chan HM, Ahmad R, Lurie S, Dandekar A, Gradziel TM, Crisosto CH (2012) High density SNP mapping and QTL analysis for fruit quality characteristics in peach (*Prunus persica* L.). Tree Genet Genomes. doi:10.1007/s11295-012-0522-7

[CR35] Morgante M, Salamini F (2003). From plant genomics to breeding practice. Curr Opin Biotechnol.

[CR36] Ogundiwin EA, Peace CP, Dandekar AM, Bliss FA, Gradziel TM, Crisosto CH (2007). Molecular genetic dissection of chilling injury in peach fruit. Acta Hortic.

[CR37] Ogundiwin EA, Marti C, Forment J, Pons C, Granell A, Gradziel TM, Peace CP, Crisosto CH (2008). Development of ChillPeach genomic tools and identification of cold-responsive genes in peach fruit. Plant Mol Biol.

[CR38] Ogundiwin EA, Peace CP, Gradziel TM, Parfitt DE, Bliss FA, Crisosto CH (2009). A fruit quality gene map of Prunus. BMC Genomics.

[CR39] Peace CP, Crisosto CH, Gradziel TM (2005). Endopolygalacturonase: a candidate gene for freestone and melting flesh in peach. Mol Breed.

[CR40] Peace CP, Crisosto CH, Garner DT, Dandekar AM, Gradziel TM, Bliss FA (2006). Genetic control of internal breakdown in peach. Acta Hortic.

[CR41] Pritchard J, Stephens M, Donnelly P (2000). Inference of population structure using multilocus genotype data. Genetics.

[CR42] Rafalski J (2010). Association genetics in crop improvement. Curr Opin Plant Biol.

[CR43] Riedelsheimer C, Lisec J, Czedik-Eysenberg A, Sulpice R, Flis A, Grieder C, Altmann T, Stitt M, Willmitzer L, Melchinger AE (2012). Genome-wide association mapping of leaf metabolic profiles for dissecting complex traits in maize. Proc Natl Acad Sci USA.

[CR44] Risch N, Merikangas K (1996). The future of genetic studies of complex human diseases. Science.

[CR45] Shulaev V, Korban SS, Sosinski B, Abbott AG, Aldwinckle HS, Folta KM, Lezzoni A, Main D, Arus P, Dandekar AM, Lewers K, Brown SK, Davis TM, Gardiner SE, Potter D, Veilleux RE (2008). Multiple models for Rosaceae genomics. Plant Physiol.

[CR46] Tommasini L, Schnurbusch T, Fossati D, Mascher F, Keller B (2007). Association mapping of *Stagonospora nodorum* blotch resistance in modern European winter wheat varieties. Theor Appl Genet.

[CR47] Verde I, Bassil N, Scalabrin S, Gilmore B, Lawley CT, Gasic K, Micheletti D, Rosyara UR (2012). Development and evaluation of a 9 K SNP array for peach by internationally coordinated SNP detection and validation in breeding germplasm. PLoS One.

[CR48] Xu Y, Crouch J (2008). Marker-assisted selection in plant breeding: from publications to practice. Crop Sci.

[CR49] Yu J, Buckler E (2006). Genetic association mapping and genome organization of maize. Curr Opin Biotechnol.

[CR50] Yu H, Xie W, Wang J, Xing Y, Xu C, Li X, Xiao J, Zhang Q (2011). Gains in QTL detection using an ultra-high density snp map based on population sequencing relative to traditional RFLP/SSR markers. PLoS One.

[CR51] Zhao K, Tung CW, Eizenga GC, Wright MH, Ali ML, Price AH, Norton GJ, Islam MR, Reynolds A, Mezey J, McClung AM, Bustamante CD, McCouch SR (2011). Genome-wide association mapping reveals a rich genetic architecture of complex traits in *Oryza sativa*. Nat Commun.

